# Beyond the image : sense and non-sense in imaging biomarkers

**DOI:** 10.1186/1470-7330-14-S1-O33

**Published:** 2014-10-09

**Authors:** Matthijs Oudkerk

**Affiliations:** 1 Department of Radiology, University of Groningen, Netherlands

## 

A biomarker or biological marker is defined as an indicator of a biological state or existence in the past of a particular type of organism. Biomarkers are *objectively measured and evaluated* as indicators of normal biological processes, pathogenic processes, or pharmacologic responses to a therapeutic intervention. Over the last two decades such biomarkers have been developed in many fields of medicine : such as in cell biology where a molecule is used as a biomarker to detect and isolate a particular cell type, in genetics where a DNA sequence is associated with the cause of or susceptibility to a disease, in pharmacology where biomarkers provide a molecular impression of a biological system (cell, animal or human) such as a blood PSA protein as an indicator of prostate cancer or blood cholesterol as a risk indicator of vascular disease etc. A recent analysis on biomarker innovation shows a huge gap between the discovery of biomarkers on one hand and the development into diagnostic application on the other. Very few biomarkers progress after initial discovery and publication to clinical validation, confirmation and at the end diagnostic application due to many complex factors such as limited confirmation of initial findings by other laboratories, rare quantitative clinical validation of biomarkers by single test and multi laboratory research and very limited progress of validated biomarker to applicable molecular diagnostic tests. In pharmacology a biomarker definition working group was initiated in 2001. Ten years later several initiatives to further develop uniformity in terminology, data acquisition, data processing and post processing etc have been integrated in the QIBA group which works its way through this enormous field of developing Imaging biomarkers [1]. An Imaging Biomaker can be defined as a single quantitative unit acquired by an imaging modality and expressing morphology or function of a cell, tissue or organ, which objectively measured and evaluated as a prognostic indicator of normal, physiological and pathogenic processes. This definition should be distinguished from an image phenotype which is a set of multiple acquired quantitative measurements obtained from digital imaging data as parameters for personalized characterization of the subject examined. An imaging biomarker should be accurate, reproducible, objective, quantitative, standardizable and robust. For successful implementation an imaging biomarker has to be fast applicable, widely available and cheap. Furthermore an imaging biomarker should deliver a single parameter on which clinical decisions can be taken. In this manner imaging biomarkers can be directly compared to other non imaging biomarkers in any diagnostic management algorithm. For imaging biomarkers there is the same long development road as for non-imaging biomarkers with the same huge innovation gap. Many candidates but very few which reach the maturity to clinical application. Each imaging modality has its specific strengths and weaknesses from which in a further analysis the candidates with the best chances can be deducted and selected for further development to clinical implementation. Only in systematic development pathways this goal can be reached and imaging can come to another era beyond the image.
